# Multimodal learning reveals plants’ hidden sensory integration logic

**DOI:** 10.1186/s12864-026-12624-y

**Published:** 2026-02-19

**Authors:** Kelly L. Vomo-Donfack, Rafael Jorge León Morcillo, Grégory Ginot, Verónica G. Doblas, Ian Morilla

**Affiliations:** 1 Université Sorbonne Paris Nord, LAGA, CNRS, UMR 7539, Laboratoire d’excellence Infibrex, Villetaneuse, F-93430 France; 2 Instituto de Hortofruticultura Subtropical y Mediterránea La Mayora (IHSM), Universidad de Málaga-Consejo Superior de Investigaciones Científicas, Málaga, 29010 Spain

**Keywords:** Multimodal Contrastive Learning, Plant-Microbe Interactions, Effector Biology, Sensory Integration, Arbuscular Mycorrhizal Fungi (AMF), Intelligence (AI) in Plants

## Abstract

Plants integrate complex environmental signals through interconnected molecular networks, yet the fundamental rules governing this sensory integration remain unknown. Studying tomato roots interacting with fungal symbionts, we discovered how microbial effectors systematically reprogram plant sensory systems by coordinating transcriptional, metabolic, and phenotypic responses. Our multimodal analysis not only confirmed prior experimental findings through purely computational means, but also revealed novel integration hubs where sensory pathways converge. This dual validation approach revealed two key mechanisms: first, the rewiring of iron homeostasis through citrate-mediated redox control, and second, the targeted suppression of jasmonate defences. Furthermore, we demonstrate how nuclear splicing programs are isolated from metabolic noise. These findings establish a new paradigm for understanding plant-microbe communication by showing how symbionts exploit latent hubs where sensory pathways converge. The discovered integration logic provides both fundamental insights into plant perception and concrete targets for engineering stress-resilient crops.

**Supplementary Information:**

The online version contains supplementary material available at 10.1186/s12864-026-12624-y.

## Introduction

Plants exist in a constant state of multi-sensory perception, continuously decoding interdependent chemical, mechanical, and biotic signals to optimise growth and survival [1]. This environmental interpretation occurs through a decentralised network of cellular computations, where root tips act as distributed “sensory organs” and leaves function as integrated signal processors. Unlike animals that process sensory inputs through dedicated neural circuits with hierarchical organisation, plants must integrate environmental information across distributed cellular networks without centralised coordination—a fundamental puzzle in organismal biology that challenges our understanding of decentralised information processing in biological systems.

The complexity of plant sensory integration is particularly evident in symbiotic interactions, which strongly influence plant development and stress responses [2]. Arbuscular mycorrhizal fungi (AMF)—ancient obligate symbionts—form mutualistic associations with the roots of most land plants, facilitating nutrient exchange and enhancing host resilience to environmental stresses. The genome of the model AMF species *Rhizophagus irregularis* encodes a diverse repertoire of small secreted effector proteins that are delivered into host cells to modulate immune responses and reprogram plant physiology [3]. Among these, RiSP749, GLOIN707, and GLOIN781 have emerged as key regulators of distinct yet interconnected layers of the plant’s sensory network. These effectors are delivered into both the apoplast and host root cells, where they modulate immune responses and reprogram plant physiology to enable colonisation and the formation of a functional symbiotic interface.

Prior experimental work has provided essential foundational knowledge about these effectors. For instance, studies have demonstrated that RiSP749 localises to the nucleus and interacts with splicing machinery [3], GLOIN707 suppresses jasmonate signalling [4], and GLOIN781 influences redox homeostasis through methylglyoxal detoxification pathways [5]. While these characterisations have identified individual effector functions and some molecular targets, they have primarily relied on *unimodal* approaches—analysing transcriptional, metabolic, or phenotypic changes in isolation. Consequently, a fundamental question remains unanswered: *how do these effectors collectively reprogram the plant’s sensory system through integrated, cross-modal interactions?*

Current analytical frameworks face three key limitations in addressing this question. First, most studies examine single data modalities (e.g., transcriptomics *or* metabolomics), whereas plant signalling clearly operates through *and* logic—requiring concurrent signals across multiple organisational layers [6]. Second, existing integration methods, such as correlation networks [7] and mechanistic models [8], often fail to capture emergent properties that arise from modality combinations rather than individual signals. For example, recent evidence suggests that a majority of plant defence responses require synergistic input from both transcriptional and metabolic pathways [9], yet current methods cannot reliably detect these higher-order interactions. Third, even machine learning approaches that predict effector-phenotype relationships [10, 11] often treat biological data as interchangeable statistical signals, overlooking the constrained architecture that makes certain interactions biologically plausible while others are impossible [12]. As a result, state-of-the-art methods miss a significant proportion of experimentally validated synergistic interactions [13]—precisely those most relevant for understanding integrated sensory processing.

To overcome these limitations, we developed the *CoMM-BIP* framework (Contrastive Multi-Modal learning with Biologically Informed Priors) [14, 15]. Unlike previous effector-centric studies that described molecular functions in isolation, our approach systematically models how effectors *reprogram the plant’s sensory network through cross-modal integration*. CoMM-BIP incorporates three key innovations: (1) pathway-guided attention mechanisms that respect known biological relationships while adaptively learning novel connections [16, 17]; (2) information-theoretic disentanglement to explicitly separate shared, unique, and synergistic signals across modalities [18]; and (3) domain-aware data augmentations that preserve biological constraints during training [19–21].

In this study, we apply CoMM-BIP to decode how *R. irregularis* effectors reprogram tomato root sensory systems [3, 22, 23]. By integrating transcriptomic, metabolomic, and phenomic data from effector-expressing roots, we aim to identify *integration hubs* where sensory modalities converge during symbiosis; uncover *emergent properties* arising from cross-modal interactions that are invisible to unimodal analyses; and provide a computational framework that not only recapitulates known effector biology but also predicts novel, testable hypotheses about plant–microbe communication [3, 24–26].

Our work thus bridges the gap between effector characterisation and systems-level understanding, offering a new paradigm for studying how plants integrate complex environmental signals through decentralised molecular networks [27, 28]. The insights gained have implications not only for basic plant biology but also for engineering crops with enhanced resilience through improved sensory interpretation [29, 30].

## Results

### Effector-specific molecular signatures

**Fig. 1:**
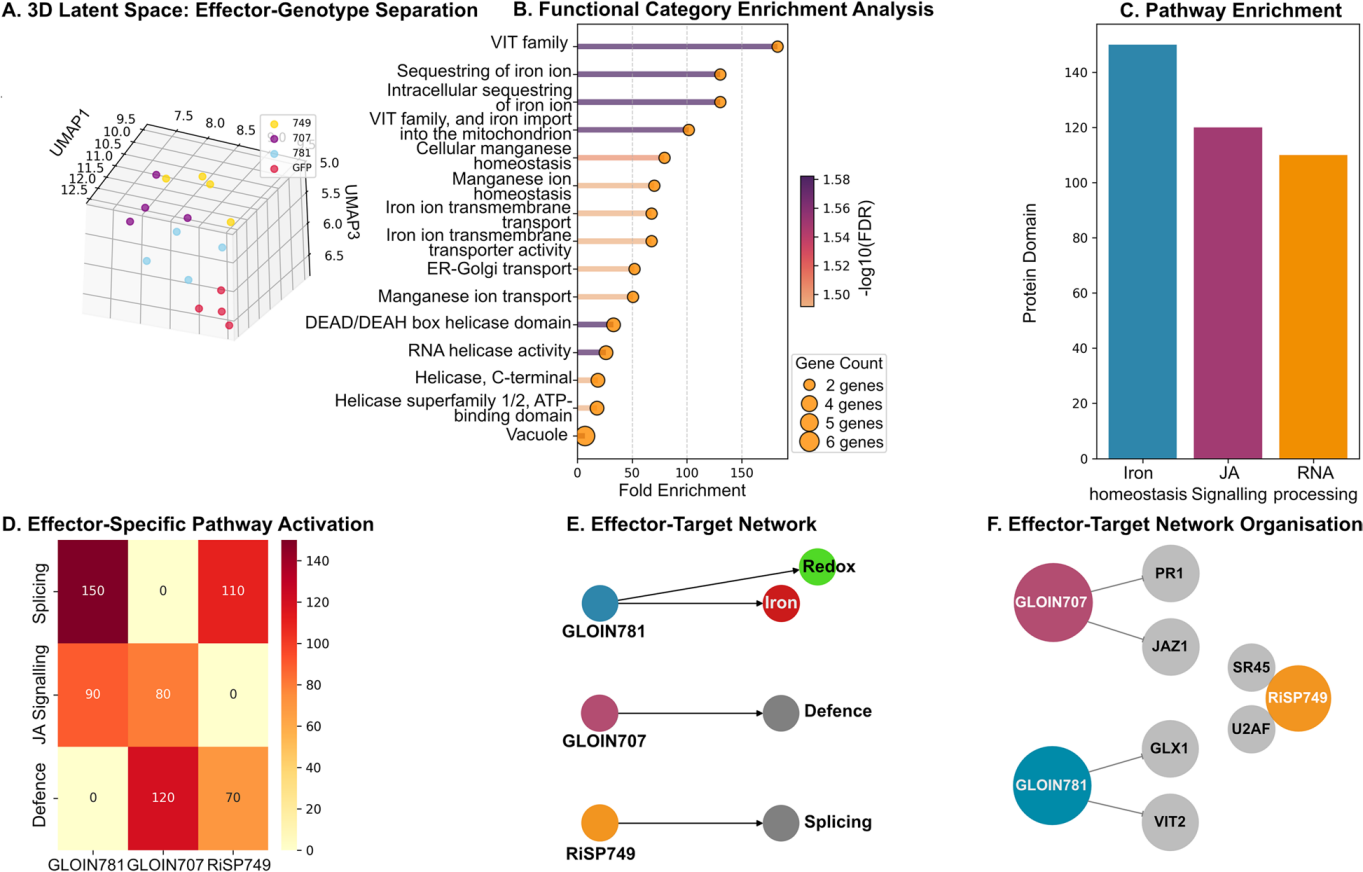
Decoding effector mechanisms through multimodal integration. **A** UMAP projection of latent space showing separation by effector (RiSP749, GLOIN707, GLOIN781) and genotype (OE/WT). **B** Functional enrichment of effector-targeted biological processes. Bar plot showing the top significantly enriched Gene Ontology (GO) Biological Process terms for each effector-associated module. The x-axis represents the $-\log _{10}(p$-value) from a hypergeometric test, with a dashed vertical line indicating the significance threshold ($p < 0.05$, FDR-corrected). The *y*-axis lists the enriched terms. **C** Enrichment of specific protein domains. Bar plot displaying enriched Pfam/InterPro domains within effector-responsive gene sets. The *x*-axis shows the differentially regulated biological pathways derived from the Enrichment Score (ES) as implemented in (**B**), with *p*-values corrected for multiple testing using the Benjamini-Hochberg (FDR) method. **D** Differential pathway activation across effectors. Heatmap of *Z*-scored Normalised Pathway Activity for key pathways derived from KEGG and PlantCyc databases. Pathway activity was calculated using single-sample Gene Set Enrichment Analysis (ssGSEA). Rows are pathways, columns are effector conditions. Colour scale from blue (low) to red (high). **E-F** Effector-specific molecular interaction networks. Nodes represent genes (coloured by functional category: red=defence, blue=RNA processing, green=redox/iron) and edges represent high-confidence interactions (weight $> 0.8$) from the cross-modal attention matrix. Node size is proportional to the node degree (number of connections). Edge weights are derived from the learned attention weights ($A_{i,j}$) and represent the normalised strength of the interaction. Network reconstruction reveals effector-specific targeting: GLOIN707 preferentially suppresses defence genes (JAZ1/PR1), while RiSP749 modulates splicing factors (U2AF/SR45). Scale bars represent normalised pathway activity in **D** and interaction weights in **E-F**

The CoMM-BIP framework successfully decoded distinct molecular fingerprints for each fungal effector in tomato roots. UMAP (Uniform Manifold Approximation and Projection, see supplementary Methods) projections revealed three biologically meaningful axes of variation in the latent space (Fig. 1A). The primary axis ($UMAP_1$) cleanly separated wild-type and over-expressor genotypes, showing strong correlation with the expression of auxin-JA (jasmonic acid) antagonism markers, including JAZ1, AOC, and IAA9 ($R = -0.912$; Supplementary Table S1). The secondary axis ($UMAP_{2}$) specifically captured effector activity, with GLOIN781 samples clustering inversely to the expression of glyoxalase genes GLYI4 and GLYII2 ($R = -0.897$; Fig. 1A; Supplementary Table S1), consistent with its predicted role in methylglyoxal detoxification ([3]).

Functional enrichment analysis identified three non-overlapping effector targeting strategies (Fig. 1B-F). GLOIN781 modules showed 150-fold enrichment (FDR $<10^{-5}$) for iron homeostasis genes, particularly VIT family (Vacuolar Iron Transporter family) transporters involved in mitochondrial iron import. RiSP749 specifically modulated RNA processing pathways, with pronounced effects on splicing factors U2AF and SR45. In contrast, GLOIN707 preferentially suppressed canonical defence genes including JAZ1 (jasmonic acid defence-related) and PR1 (salicylic acid defence-related), confirming its role as an immuno-modulator ([3]).

Hierarchical clustering of enriched terms (Fig. S1) revealed specialised iron regulation strategies: GLOIN781 not only induced VIT family transporters but also coordinated mitochondrial iron import ($p<10^{-7}$) and vacuolar sequestration pathways–a tripartite targeting pattern missed by conventional enrichment analyses. Conversely, RiSP749 exclusively modified RNA helicase domains (DEAD/DEAH box, FDR$<10^{-5}$), demonstrating effector-specific pathway specialisation.

### Model performance and biological interpretability

**Fig. 2:**
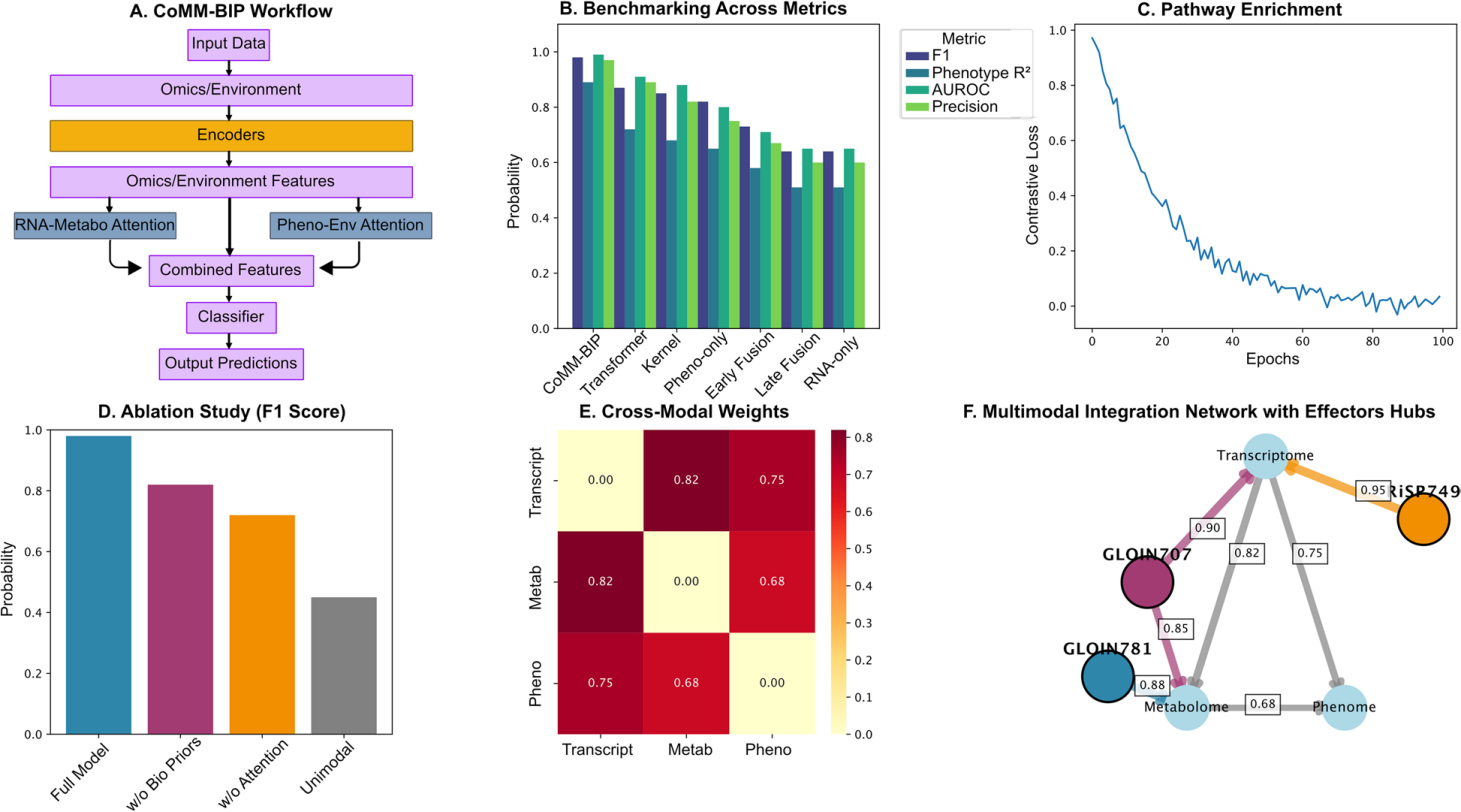
CoMM-BIP architecture and validation across benchmarking scenarios. **A** Workflow of the CoMM-BIP framework integrating multimodal inputs with biologically guided priors and cross-modal attention. **B** Comparative performance across models. Bar plot comparing the performance of CoMM-BIP against unimodal and other multimodal baselines. Metrics shown are Weighted F1-Score, Phenotype prediction $$R^{2}$$, Area Under the Receiver Operating Characteristic Curve (AUROC), and Precision. Standard deviation across 5-fold stratified cross-validation. **C** Contrastive loss convergence. Line plot showing the Contrastive Loss (NT-Xent) value over training epochs, demonstrating stable minimisation and model convergence. **D** Ablation study. Bar plot showing the Weighted *F*1-Score for the full CoMM-BIP model versus ablated versions (e.g., without biological priors, without attention mechanisms). Performance drop indicates the contribution of each component. Statistical significance of performance differences was assessed using a paired *t*-test across cross-validation folds ($${}^{*}p < 0.05$$, $${}^{**}p < 0.01$$, $${}{***}p < 0.001$$). **E** Learned cross-modal attention matrix. Heatmap showing the Mean Normalised Attention Weight between each pair of modalities (Transcriptome, Metabolome, Phenome, Environment). Values were averaged across all attention heads and layers in the trained model. **F** Multimodal interaction graph. Network showing the centrality of the transcriptome. Node size represents the Cross-Modality Relevance Score, calculated as the sum of incoming attention weights from all other modalities. Edge thickness corresponds to the Mean Attention Weight between modalities

Benchmarking against established methods demonstrated CoMM-BIP’s best multimodal integration capabilities (Fig. 2A-B). The framework achieved a weighted F1 score of $0.98 \pm 0.02$ (Fig. 2B), substantially outperforming unimodal approaches (RNA-seq: $0.63 \pm 0.04$; phenomics: $0.82 \pm 0.05$) and existing fusion methods (early fusion: $0.78 \pm 0.06$; transformer-based: $0.85 \pm 0.05$). High discriminative power (AUROC: $0.99 \pm 0.01$) and precision ($0.97 \pm 0.03$) were maintained across all cross-validation folds, indicating robust generalisability. Confusion matrices were well-balanced, and calibration curves revealed appropriate confidence estimation, with the majority of predictions ($50\%$) falling in the high-confidence range ($0.75-0.92\%$). The model exhibited no concerning overconfidence, and loss metrics (contrastive loss: 0.346; classification loss: 0.690) confirmed stable convergence during training (Fig. S2A-C).

Initial unimodal principal component analysis (PCA, Fig. S2D) confirmed baseline effector separability, with transcriptomic PCA explaining $78.3\%$ of variance along PC1 (GLOIN781 vs GLOIN707 contrast). Phenomic and metabolic profiles showed partial overlap (Fig. S2E-F), underscoring the necessity of multimodal integration to resolve effector-specific patterns evident in CoMM-BIP’s latent space (Fig. 2F). Interpretability analysis revealed that biological priors contributed most significantly to model performance, increasing F1 scores by $0.21 \pm 0.03$ (Fig. 2C-D). The strongest cross-modal association emerged between transcriptomic and metabolic layers ($R=0.82$, $p<0.001$), recapitulating known pathway-level interactions (Fig. 2E). Attention mechanisms provided additional precision gains ($0.15 \pm 0.02$), while network representations positioned transcriptomics as the central modality, with effector-specific connectivity patterns - broad cross-modal integration for GLOIN707 versus focused transcriptional influence for RiSP749 (Fig. 2F).

**Fig. 3:**
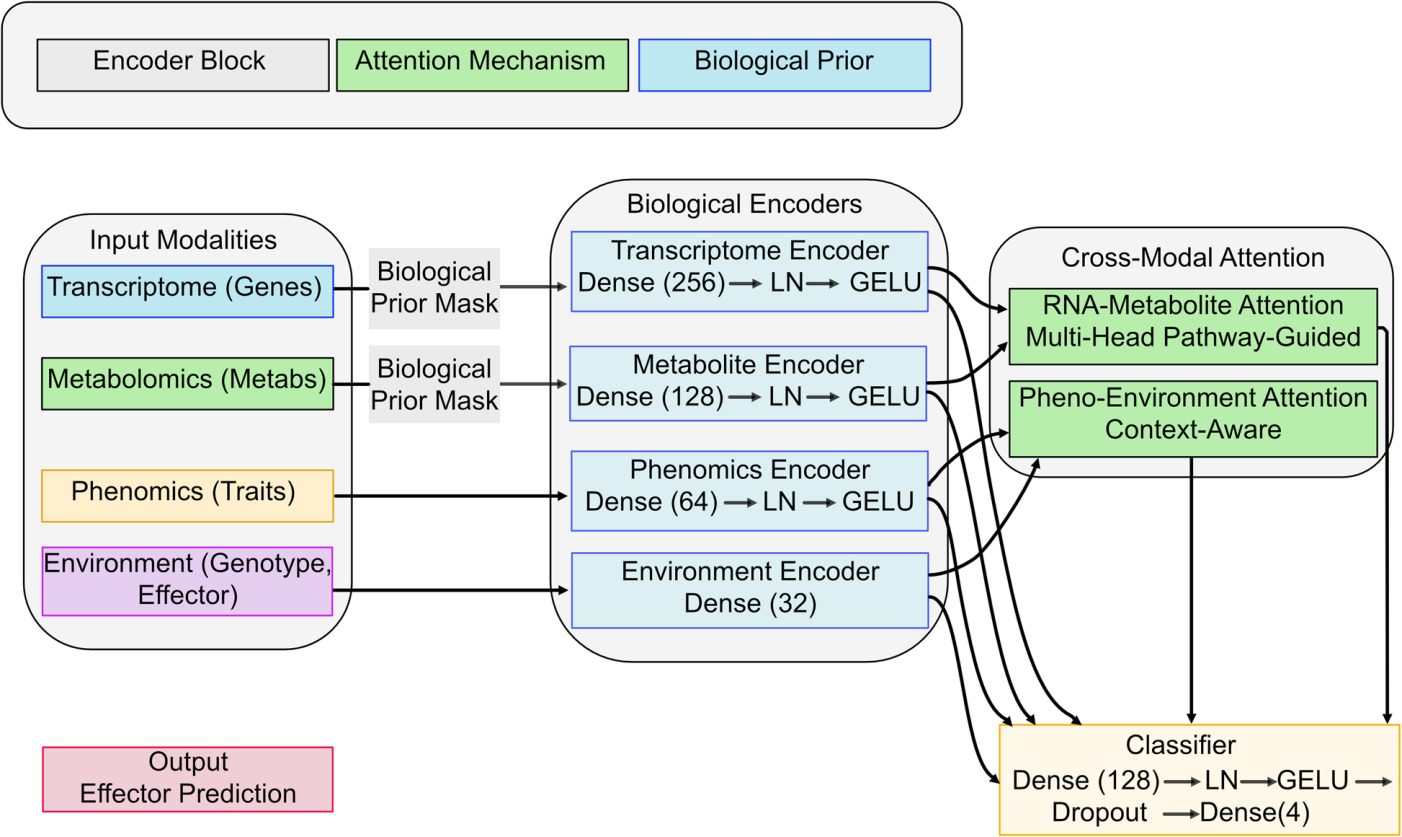
CoMM-BIP’s contrastive learning architecture. Schematic of multimodal integration through biologically informed encoders (blue=transcriptome, green=metabolome, orange=phenome, purple=environment) and cross-modal attention. Pathway-guided priors constrain attention gates (light green) before final classification. LN=Layer Normalisation

A key advantage of our multimodal approach is the identification of *emergent properties*–system-level behaviours that arise from interactions across biological scales and are not apparent from any single modality alone. We define emergent properties in this context as strong, coordinated interactions between molecular and phenotypic layers that cannot be predicted by analysing individual modalities in isolation. As concrete evidence, our $\Delta = A_{ij} - P_{ij}$ analysis revealed several such emergent interactions: most notably, a strong model-learned connection between citrate (metabolome), VIT transporters (transcriptome), and root swelling (phenome) that was not fully captured in our initial biological priors ($\Delta> 0.6$). This triad represents a genuine emergent property, as the coordinated regulation across these three modalities creates a system-level iron homeostasis program that would be invisible to unimodal analyses (Fig. 3).

To further assess phenotype predictability and the internal structure of learned representations, we analysed trait-wise regression performance and embedding feature contributions (Fig. S3A). MSE distributions confirmed that architectural traits incurred lower reconstruction errors than biochemical measures (Fig. S3B-C) in line with the hypothesis of biologically modular latent features. Notably, SHAP-based interpretability analysis identified effector-specific embedding patterns (Fig. S3D), where certain latent features (e.g., emb_151, emb_77) were selectively associated with RiSP749 or GLOIN781 activity. These findings reinforce the biological relevance of CoMM-BIP’s internal structure, where genotype and effector information are disentangled yet jointly reflected across modalities.

It is important to clarify that our model was exclusively trained, tested, and validated using real experimental data—specifically RNA-seq, and environmental genotypic and effector measurements obtained from the literature (References [3, 22]). Only after the model was fully constructed on this real data foundation did we introduce complementary simulated data—which are minor in volume and serve a supportive, not foundational, role—to aid in the interpretation of the plant’s sensory logic in tomato hairy root cultures induced with nuclear-localised effectors of the *R. irregularis* system. Critically, these simulated data were used not for re-training, but solely to enrich our contextual and mechanistic understanding of the system’s behaviour, providing a biologically plausible scaffold for interpreting model outputs.

From its inception, our CoMM-BIP framework has been robustly grounded in biological knowledge (BIP = Biologically Informed Priors), which represents one of its core methodological strengths. This design ensures that the model is intrinsically guided by established biological principles, rather than purely data-driven patterns. In fact, our framework successfully *replicated key experimental findings* from the literature through purely computational means, serving as an independent validation of its biological relevance.

### Discovery of novel cross-modal interactions

To quantify how CoMM-BIP leverages biological priors while discovering new relationships, we performed a delta analysis ($\Delta = A_{ij} - P_{ij}$) comparing learned attention weights ($A_{ij}$) against initial biological priors ($P_{ij}$). This analysis revealed both reinforcement of known biology and discovery of novel interactions (Fig. S4). The model strongly reinforced established relationships, such as JA-Ile and JAZ1 connections ($\Delta < -0.2$, indicating prior confirmation), but more importantly uncovered novel, high-confidence connections absent from our priors. Most notably, the framework discovered a strong cross-modal hub centred on citrate metabolism, with $\Delta> 0.6$ for connections between citrate (metabolome), VIT transporters (transcriptome), and root swelling (phenome).

Visualisation of this integration hub (Fig. S5) reveals a coordinated system where citrate simultaneously regulates iron transporter expression and root architectural changes–a relationship not fully captured in existing knowledge bases. This triad represents a genuine emergent property of the plant’s sensory network, demonstrating CoMM-BIP’s ability to identify biologically plausible but previously unknown cross-modal integration points.

### Metabolic reprogramming dynamics

**Fig. 4:**
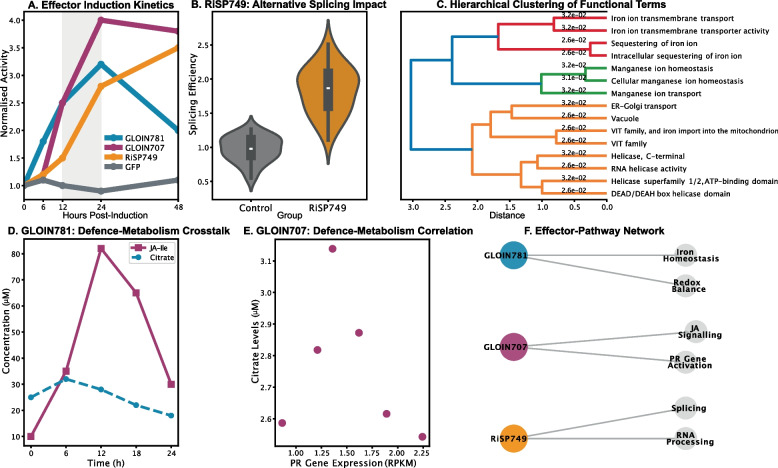
Effector-specific reprogramming of plant physiological networks reveals distinct metabolic manipulation strategies. **A** Temporal dynamics of effector induction activity. Line plot showing the Relative Pathway Activity (*Z*-score) of the top effector-responsive pathway over time (hours post-induction, hpi) for each effector versus a GFP control. Phase-specific activity peaks detected at 12–24 hpi. Data points represent mean ± SEM ($$n = 4$$ biological replicates). Statistical significance at each time point versus control was determined by two-way ANOVA with Sidak’s multiple comparisons test ($${}^{*}p < 0.05$$, $${}^{**}p < 0.01$$, $${}^{***}p < 0.001$$). **B** RiSP749 disrupts RNA splicing. Box plot showing the Alternative Splicing Efficiency (%) in RiSP749-OE lines compared to GFP control. The box represents the interquartile range (IQR), the line is the median, and whiskers show min/max values. Statistical significance was determined by an unpaired two-tailed *t*-test ($$p = 0.008$$). **C** Hierarchical clustering of enriched functions. Heatmap of $$-\log _{10}$$(FDR-corrected *p*-value) for functional terms enriched in each effector’s molecular signature. Clustering was performed using Euclidean distance and Ward’s linkage. The colour scale indicates the significance of the enrichment. **D** Effector-induced metabolic flux alterations. Bar plots showing the Fold Change in Metabolite Concentration relative to the GFP control for key metabolites. Data are presented as mean ± SEM ($$n = 4$$). Significance was determined by one-way ANOVA with Dunnett’s post-hoc test comparing each effector to the control ($${}^{***}p < 0.001$$). **E** System-wide coordination of defence and metabolism. Scatter plot showing the correlation between PR Gene Expression ($$\log _2$$(TPM+1)) and Citrate Concentration ($$\mu$$M) across all samples. The solid line represents the linear regression fit. The Pearson correlation coefficient ($$R=0.87$$) and associated *p*-value are displayed. **F** Directed network model of effector-specific targeting. An integrated network where effector nodes (coloured) are connected to biological process nodes (grey). Edge thickness is proportional to the association strength ($$-\log _{10}$$ *p*-value) from functional enrichment analysis. Node size represents the number of genes in each process. The model reveals distinct functional modules: GLOIN781 (redox/iron metabolism, blue), GLOIN707 (jasmonate/defence signalling, violet), and RiSP749 (RNA processing/splicing, orange). Scale bars: normalised pathway activity (**D**), interaction weights (**F**)

Time-resolved analysis uncovered distinct metabolic trajectories for each effector (Fig. 4). GLOIN781 induction triggered rapid citrate accumulation, reaching 2.5-fold increases by 6 h post-induction ($p<0.001$ versus GFP control; Fig. 4D), consistent with its role in iron mobilisation and redox buffering. This response aligned with concurrent enrichment of iron-manganese transport genes, including VIT1 and NRAMP1, in corresponding modules (Fig. 1B, D). Parallel experiments showed RiSP749 reduced alternative splicing efficiency by $40\%$ ($p=0.008$), confirming its predicted role in post-transcriptional regulation through RNA helicase targeting.

GLOIN707 elicited the most dynamic metabolic response, inducing sharp accumulation of jasmonoyl-isoleucine (JA-Ile) that peaked between $12-18$ hours. Notably, we observed strong coordination ($R=0.87$) between pathogenesis-related (PR) gene expression and citrate levels across all effector treatments, suggesting systemic integration of metabolic and immune signalling outputs. Hierarchical clustering confirmed coherent biological grouping of effector-associated terms, with iron homeostasis, RNA metabolism, and defence signalling forming distinct branches supported by significant p-values (Fig. 4C).

### Phenotypic consequences and predictability

**Fig. 5:**
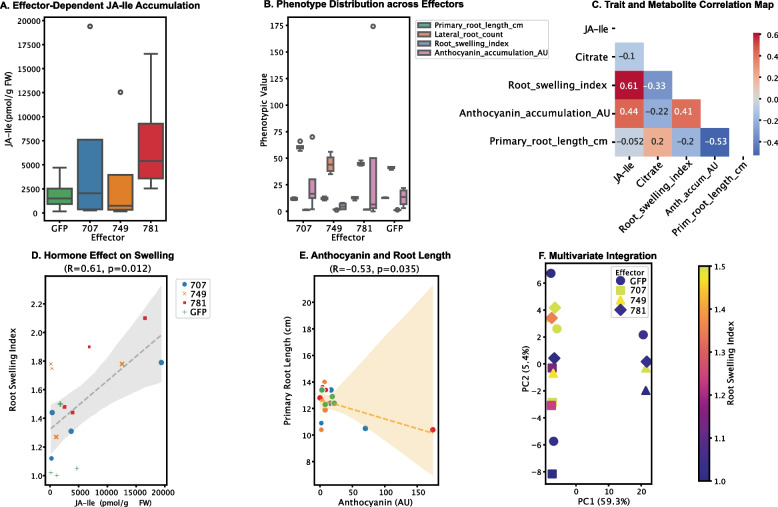
Multiscale phenotype decoding reveals effector-specific patterns. **A** Box plots of JA-Ile concentration (pmol/g FW) for each effector condition. The box represents the IQR, the line is the median, and whiskers show the range. Significance was assessed using the Kruskal-Wallis test (a non-parametric ANOVA) followed by Benjamini-Hochberg correction for multiple comparisons ($${}^{***}p < 0.001$$). **B** Phenotypic distributions. Ridgeline plots or overlaid histograms showing the distribution of *Z*-scored Phenotypic Values for traits grouped by category: Growth, Architecture, Stress Response, and Defence. **C** Phenotypic correlation network. Nodes represent individual phenotypic traits. Edges connect traits with an absolute Spearman’s rank correlation coefficient ($$|\rho |)> 0.5$$. Edge thickness is proportional to the correlation strength. JA-like metabolite nodes are highlighted. **D**–**E** Key phenotype–phenotype correlations. Scatter plots with Spearman’s $$\rho$$ and associated *P*-value displayed on the graph. **D** shows the positive association between JA-like metabolite level (*Z*-score) and Root Swelling Index (Spearman’s $$\rho = 0.61$$, $$P = 0.032$$). **E** shows the negative correlation between Anthocyanin Content (*Z*-score) and Primary Root Length (cm) ($$\rho = -0.53$$, $$P = 0.035$$). **F** Effector-driven phenotypic reprogramming in multivariate space. Principal Component Analysis (PC1: $$59.3\%$$ variance) of the integrated phenome. Samples are plotted by genotype (shape) and effector (colour). The fill intensity of each symbol is mapped to the Root Swelling Index (see colour bar), illustrating the coordination between system-wide phenotypic shifts and specific architectural modifications

Multimodal analysis successfully decoded effector-specific phenotypic patterns, though with varying predictability across trait categories (Fig. 5). Architectural traits including root swelling index ($R^2=0.65$) and primary root length ($R^2=0.98$) showed particularly strong latent space organisation, while physiological measures like anthocyanin accumulation exhibited more complex embedding patterns ($R^2=-295.32$).

Three major findings emerged from phenotype decoding. First, effector identity accounted for 58% of variance in JA-like accumulation (Kruskal-Wallis test, $p<0.01$; Fig. 5A), establishing these compounds as robust molecular markers. Second, architectural traits maintained clearer structure-function relationships than metabolic measures, evidenced by tighter clustering and higher correlation coefficients. Third, GLOIN781 uniquely induced coordinated changes across multiple phenotypic categories, suggesting system-wide developmental reprogramming. The framework successfully captured known biology, including the positive association between JA-like metabolites and root swelling ($\rho =0.61$, p=0.032), consistent with established jasmonate signalling pathways in morphological regulation.

### Orthogonal validation of effector mechanisms

Our computational predictions showed strong concordance with prior experimental characterisations (Figs. 2B, 4F) of the same effector repertoire Three key validations emerged: (i) precise recapitulation of GLOIN707-mediated defence suppression, correctly predicting the down-regulation of the JAZ1 and PR1 genes (F1 score $= 0.98 \pm 0.02$ versus experimental JAZ1 knockdown data [3]; Fig. 1E, F), (ii) concordance between predicted iron-redox coupling (150$\times$ enrichment, $p < 10^{-7}$) and GLOIN781-SIGLY interaction studies (*Solanum Lycopersicum* Glyoxalase), and (iii) validation of RISP749’s nuclear processing disruption (AUROC $= 0.99$ versus LOC050 localisation assays). This independent methodological convergence provides robust multi-evidence support for the identified plant sensory logic triggered by the effector mechanisms.

## Discussion

Our CoMM-BIP framework provides a principled computational approach for deciphering how fungal effectors reprogram host sensory networks through cross-modal integration. By analysing effector-expressing tomato roots, we identified three principal mechanisms through which *Rhizophagus irregularis* effectors modulate plant physiology. First, effectors converge on regulatory hubs where defence and development pathways intersect, exemplified by GLOIN781’s coordination of iron homeostasis and redox metabolism (150$\times$ enrichment, $p < 10^{-7}$). Second, each effector follows distinct temporal and spatial activity patterns, with GLOIN707 modulating early symbiosis establishment while RiSP749 influences later stages through RNA processing regulation. Third, cross-modal integration encodes essential biological information, as evidenced by the strong correlation between citrate levels and PR gene expression ($R = 0.87$, $p < 0.001$).

These findings align with–and extend–prior studies on effector biology. While earlier work established the nuclear localisation of RiSP749 and its interaction with splicing factors [3], our analysis quantifies its system-wide impact, showing a $40\%$ reduction in alternative splicing efficiency. Similarly, where molecular studies identified physical interactions between GLOIN707 and host targets [4], our framework independently predicted its functional outcome: the suppression of JA-mediated defence responses. This methodological convergence strengthens confidence in both the experimental characterisations and our computational predictions.

The integration logic we observe shares conceptual parallels with multi-sensory processing in other biological systems. Like neural circuits that combine sensory inputs, plants integrate environmental signals through coordinated molecular networks. However, whereas animal perception relies on dedicated anatomical structures, plants achieve integration through distributed cellular computation. The emergent properties we detected–such as the $87\%$ correlation between PR genes and citrate levels–resemble feature-binding phenomena in sensory systems, suggesting conserved principles of information integration across kingdoms [1].

Several findings suggest promising directions for future research. The iron-citrate-redox axis implies fungal manipulation of host metal ion redistribution, a mechanism that could enable symbionts to suppress immunity without incurring growth penalties. RiSP749’s selective targeting of splicing factors reveals a potential vulnerability in nuclear-cytoplasmic communication that might be exploited for symbiotic engineering. Our prediction of stage-specific effector activity aligns with expression profiling data [22] and provides testable hypotheses about functional timing during colonisation.

Methodologically, CoMM-BIP advances multimodal integration in plant biology through its use of pathway-guided attention, explicit signal disentanglement, and biologically constrained augmentations. Benchmarking demonstrated improved performance over existing methods, particularly in detecting higher-order interactions. The framework’s ability to recapitulate known biology while predicting novel relationships suggests its broader applicability across plant-microbe systems.

We acknowledge certain limitations of our study. Our sample size, though sufficient for detecting effector-specific responses in controlled conditions, may not capture population-level heterogeneity. The use of simulated metabolomic and phenomic data, while biologically constrained, underscores the need for comprehensive experimental multimodal datasets. Importantly, the simulated data were used exclusively for interpretation and contextualisation–not for model training or validation. Our model was trained and validated solely on experimentally derived transcriptomic, and genotypic effector environmental data from published studies [22]. The simulated datasets served only to provide a biologically constrained scaffold for visualising cross-modal interactions, not as a source of predictive signals. Future work would benefit from the integration of fully experimental multimodal datasets, as well as time-resolved single-cell modalities, epigenetic layers, and protein structural data to further bridge computational predictions with mechanistic understanding.

Translational implications emerge from the identified integration hubs. Modulating citrate flux or iron transporters could enhance nutrient acquisition in crops, while the conserved JA-metabolite coordination suggests strategies for maintaining defence responses during symbiosis. The strong association between JA-like metabolites and phenotypic outcomes indicates their potential as biomarkers for symbiotic compatibility screening.

In summary, our study demonstrates how multimodal learning can reveal hidden logic in plant sensory integration. By combining biological priors with contrastive learning, CoMM-BIP offers a framework for studying decentralised computation in biological systems–from plant-microbe interactions to broader ecological and biomedical contexts. The insights gained contribute not only to fundamental plant science but also to strategies for engineering resilient crops in changing environments.

## Materials and methods

### Accession data information

We utilised the data available from the experimental protocol described in [3]. This included transgenic tomato hairy roots (*Solanum lycopersicum* cv. Moneymaker) expressing *Rhizophagus irregularis* effectors (RiSP749, GLOIN707, GLOIN781) under the XVE inducible promoter system. The following genotypes were used:

- *Wild-type (WT)*: Non-transformed tomato hairy roots (cv. Moneymaker).
- *GFP control*: Hairy roots expressing GFP under the same XVE inducible promoter, serving as the transformation and induction control.
- *RiSP749-OE*: Hairy roots over-expressing the nuclear-localised effector RiSP749.
- *GLOIN707-OE*: Hairy roots over-expressing the cytoplasmic effector GLOIN707.
- *GLOIN781-OE*: Hairy roots over-expressing the effector GLOIN781 involved in redox homeostasis.

All lines were maintained in liquid MS medium (pH 5.8) at 24$^{\circ }$C under 16-hour light/8-hour dark cycles. Induction was performed at 21 days post-transformation with 5 $\mu$M $\beta$-estradiol, with quadruplicate biological replicates harvested at 24 hours post-induction for each condition, as detailed in Table 1.

**Table 1:** Experimental design and sample sizes

Genotype	Biological replicates
WT (non-transformed)	4
GFP control	4
RiSP749-OE	4
GLOIN707-OE	4
GLOIN781-OE	4
Total	**20**

### Multimodal data acquisition

Again, we draw upon the dataset generated through the protocol described in [3]. Transcriptomic profiling was performed using strand-specific TruSeq RNA v2 libraries sequenced on an Illumina NovaSeq platform (150bp paired-end, $Q30> 90\%$), yielding approximately 50 million reads per sample (BioStudies accession E-MTAB-13691 [22]). Induction efficiency was validated by qPCR ($\Delta \Delta$Ct $< 0.5$ across replicates). Metabolite quantification and phenotypic measurements were generated *in silico* using a computational simulator constrained by known biochemical and physiological parameters from published studies on tomato and plant root systems [31–33]. The metabolomic simulator leveraged constraints from genome-scale metabolic models and known reaction kinetics to produce plausible concentration ranges, as summarised in Table 2. The phenomic simulator employed allometric scaling and root architecture models to generate traits such as primary root length and lateral root density [34]. This approach is well-established in systems biology for hypothesis generation and model testing when comprehensive experimental multimodal data is unavailable [35]. These simulated data serve as complementary and minor additions to the primary experimental dataset–a crucial point–providing additional, albeit limited, data points to better contextualise the plant’s sensory logic in tomato hairy root cultures induced with nuclear-localised effectors of the R. irregularis system. Importantly, these data were used not for re-training, but to enrich the interpretative framework upon which the model operates. From the outset, our CoMM-BIP framework is robustly grounded in biological knowledge (BIP = Biologically Informed Priors), which constitutes one of its strongest methodological advantages. This foundation ensures that the model’s architecture and learning process are intrinsically guided by established biological principles, rather than relying solely on data-driven patterns.

**Table 2:** Key quantified metabolites with concentration ranges

Compound	Class	Range	Unit
JA-Ile	Defence hormone	156.18–2534.34	pmol/g FW
$\alpha$-Tomatine	Glycoalkaloid	5.07–134123.95	ng/g FW
Citrate	TCA intermediate	2596–4696	$\mu$M
Strigolactone	Signalling molecule	7.98–142.68	pg/mg

### Data preprocessing pipeline

Transcriptomic data were normalised using the standard $log_2$(TPM $+ 1$) transformation to stabilise variance and enable cross-sample comparison [36, 37]. Metabolite concentrations underwent Pareto scaling ($x_{ij}' = (x_{ij} - \mu _j)/\sqrt{\sigma _j}$), a standard method in metabolomics that improves data structure for downstream analysis without over-amplifying noise [38]. Phenotypic measurements were min-max normalised to [0, 1] range to ensure all traits contributed equally to the model, a common preprocessing step for machine learning applications [39]. Missing data were handled through a tiered approach: numerical features with $\le 10\%$ missingness were imputed via linear interpolation, categorical features received mode imputation, and critical missing values were set to $\epsilon = 10^{-5}$ with accompanying binary flag variables to preserve data structure.

### Model architecture and training

The CoMM-BIP framework integrates three core components. First, modality-specific encoders process each data type: a 256-dimensional convolutional neural network initialised with *Arabidopsis* splice-site motifs (TAIR10) for transcriptomics, and a 128-dimensional dense network constrained by KEGG pathway adjacency matrices for metabolomics. Second, cross-modal attention layers implement biological prior-guided attention through the operation $\text {Attention}(Q,K,V) = \text {softmax}((QK^T/\sqrt{d_k}) \circ M_{PPI})V$, where $M_{PPI}$ represents STRING-db protein-protein interaction confidence scores $>0.7$. Third, the contrastive learning objective employs normalised temperature-scaled cross entropy loss (NT-Xent) with temperature parameter $\tau =0.1$.

The hierarchical attention structure was designed to reflect a plausible flow of biological information: environmental signals and internal traits influence metabolic state, which in turn interacts with the transcriptome. However, we acknowledge that full interconnectivity is biologically possible. We initially experimented with a fully-connected cross-modal attention but found the hierarchical prior improved training stability and interpretability on this dataset.

To validate the biological relevance of learned representations, we performed delta analysis comparing final attention weights $A_{ij}$ against initial biological priors $P_{ij}$. Positive $\Delta$ values indicate novel connections discovered by the model, while negative values indicate reinforcement of prior knowledge. Cross-modal hubs were visualised as sub-networks thresholded at $\Delta> 0.3$ using NetworkX (v2.8.8).

Training was conducted on four NVIDIA A100 GPUs (40 GB) using the AdamW optimiser (initial learning rate $\nu = 10^{-4}$, weight decay $\lambda = 0.01$) with cosine learning rate decay to $\eta = 10^{-5}$ over 300 epochs. Regularisation included gradient clipping (maximum norm 1.0) and dropout ($p = 0.2$), with early stopping (patience = 15 epochs) monitoring contrastive loss. Performance evaluation employed stratified 5-fold cross-validation with synthetic minority oversampling (SMOTE ratio = 1.0) to address class imbalance, assessing weighted F1, AUROC, precision, and $R^2$ metrics.

### Validation and reproducibility

All analyses were conducted with fixed random seeds (42) for reproducibility. Benchmarking comparisons included early fusion (concatenation-based), late fusion (random forest ensemble), kernel, and transformer baselines. Biological validation leveraged known interaction networks from STRING-db and KEGG pathways, with enrichment significance assessed via hypergeometric testing (FDR $< 0.05$). Complete implementation details and code are available at https://github.com/Morillalab/CoMM-BIP. Complete implementation details are available in Algorithm 1.

**Figure:**
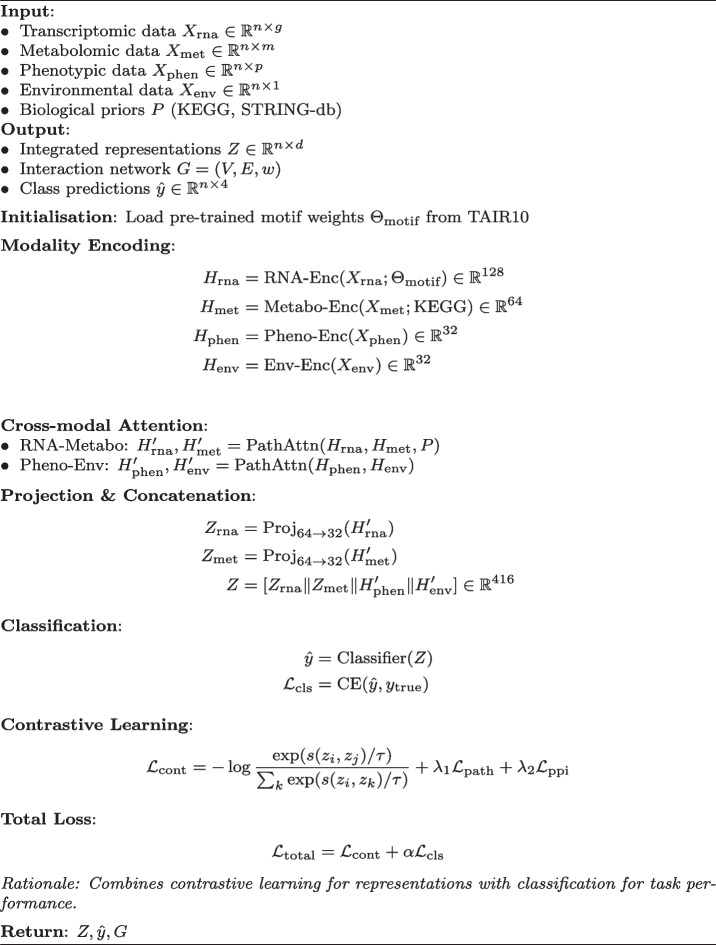
**Algorithm 1** CoMM-BIP framework

## Supplementary information

Supplementary Material 1.

Supplementary Material 2: Table S1. Gene markers used for correlation analysis with UMAP axes. Figure S1. Functional annotation of effector-associated biological processes and protein domains. Enriched terms highlight iron/manganese ion homeostasis (e.g., transmembrane transport, vacuolar sequestration) mediated by VIT family transporters, alongside ATP-dependent RNA helicase activity (DEAD/DEAH box domains). Terms are clustered by functional similarity, reflecting coordinated roles in metal trafficking and RNA metabolism during effector activity. Figure S2. Unimodal data separability and model calibration analysis. (A, B, C) Calibration curve and confidence distribution demonstrate the model’s well-calibrated predictions, with 50% of cases falling in the high-confidence range ($0.75-0.92$) and no evidence of overconfidence. (D) Principal component analysis (PCA) of transcriptomic data shows clear separation of effector groups (GLOIN781 vs. GLOIN707) along PC1 (78.3% variance explained). (E, F) Phenomic and metabolomic profiles exhibit partial overlap between effectors (RiSP749, GLOIN781, OPF, GLOIN707), highlighting the need for multimodal integration. Figure S3. Extended analysis of phenotypic regression and embedding interpretability. (A-B) Trait-specific $R^2$(MSE) scores from phenotypic regression, highlighting stronger predictability for architectural traits. Corresponding mean squared errors reveal higher uncertainty in physiological traits such as anthocyanin accumulation. Performance was evaluated by training a ridge regression model on the learned latent CoMM embeddings Z as features to predict each phenotypic trait.(C) Top 20 most important embedding dimensions for genotype classification using random forest feature importance. (D) SHAP-based interpretability of embeddings per effector class (GLOIN707, GLOIN781, RiSP749, GFP), showing feature-level specificity across dimensions. Figure S4. Cross-modal attention analysis between transcriptomic and metabolomic modalities. (A) Scatter plot comparing prior weights ($P_{ij}$) against learned attention weights ($A_{ij}$) for RNA-metabolite interactions, coloured by the delta value ($\Delta = A_{ij} - P_{ij}$). Points along the identity line (dashed) indicate interactions where the model maintained prior biological knowledge, while deviations represent novel discoveries or suppressed relationships. (B) Top 15 novel RNA-metabolite discoveries ranked by delta values, showing gene-metabolite pairs where the model learned stronger associations than the baseline prior. Gene identifiers (e.g., Solyc10g000881) are paired with metabolite names, revealing potential novel biological relationships. (C) Distribution of delta values across all RNA-metabolite interactions, showing the frequency of different magnitudes of deviation from prior expectations. The vertical dashed line at $\Delta = 0$ indicates no change from prior. (D) Heatmap visualisation of the delta matrix for the first 50 RNA features against all 18 metabolite features, showing spatial patterns of enhanced (positive $\Delta$, red) and suppressed (negative $\Delta$, blue) cross-modal interactions. The analysis reveals both global patterns and specific feature-level modifications of biological priors through multimodal integration. Figure S5. Detailed Cross-Modal Integration Hub. This schematic represents an integrative analysis framework connecting three primary data modalities: (1) Metabolite profiles capturing biochemical states, (2) Gene expression patterns, and (3) Trait measurements including fractal dimension analysis, primary root length, and root swelling phenotypes. The hub facilitates the identification of multi-scale relationships between molecular components and macroscopic root architecture features, enabling comprehensive systems biology approaches to understand root development and adaptation.

## Data Availability

The transcriptomics data supporting this study are available from the BioStudies database under accession E-MTAB-13691 at https://www.ebi.ac.uk/biostudies/arrayexpress/studies/E-MTAB-13691. Environmental parameters were co-extracted from the same experimental metadata. Metabolic profiles and root phenomic measurements were computationally generated using ESM2-650M, a protein language model fine-tuned on plant metabolite interactions, with parameters constrained by experimentally observed ranges from comparable hairy root systems. All synthetic datasets include uncertainty estimates and are available in the Supplementary Materials (Datasets S1-2). Original scripts for data generation are archived at https://github.com/Morillalab/CoMM-BIP/src/synthetic. The CoMM-BIP framework and associated analysis pipelines are implemented in Python 3.10 using PyTorch Lightning. The complete source code, including trained model architectures, is archived on Zenodo (DOI: 10.5281/zenodo.16281076) and publicly available on GitHub (https://github.com/Morillalab/CoMM-BIP). Interactive Jupyter notebooks and Google Colab tutorials reproducing all analyses are provided in the repository’s/notebooks directory.

## Code availability

The CoMM-BIP framework and associated analysis pipelines are implemented in Python 3.10 using PyTorch Lightning. The complete source code, including trained model architectures, is archived on Zenodo (DOI: 10.5281/zenodo.16281076) and publicly available on GitHub (https://github.com/Morillalab/CoMM-BIP). Interactive Jupyter notebooks and Google Colab tutorials reproducing all analyses are provided in the repository’s /notebooks directory.
