# Supplementary material for: Multimodal learning reveals plants’ hidden sensory integration logic
Source: BMC Genomics. 2026 Feb 19;27:312. doi: 10.1186/s12864-026-12624-y (PMC13032346; doi:10.1186/s12864-026-12624-y)
Supplement: Supplementary file 1 — Supplementary Material 1. [file 12864_2026_12624_MOESM1_ESM.zip › Vomo_Morilla_et_al_supplementary.pdf]

Supplementary Methods for: "Multimodal Learning Reveals Plants' Hidden Sensory Integration Logic"

Kelly L. Vomo-Donfack et al.

This document provides a comprehensive description of the computational methods and analytical procedures used in the CoMM-BIP framework, supplementing the high-level overview in the main text. All analyses were implemented in Python and executed in Google Colab.

Data Preprocessing and Normalisation

Transcriptomic Data

Gene-level TPM values obtained from E-MTAB-13691 were processed using pandas (v1.5.3) and NumPy (v1.22.4). Normalisation was applied using:

$$X_{\text{norm}} = \log_2(\text{TPM} + 1)$$

This transformation stabilizes variance across the wide dynamic range of gene expression values [1].

Metabolomic Data

The *in silico* metabolite concentrations were preprocessed using scikit-learn (v1.1.0). Pareto scaling was applied to each metabolite j across all samples i:

$$x'_{ij} = \frac{x_{ij} - \mu_j}{\sqrt{\sigma_j}}$$

where μ_j is the mean and σ_j is the standard deviation of metabolite j.

Phenotypic Data

All phenotypic measurements were min-max normalised to a $[0, 1]$ range using scikit-learn's `MinMaxScaler`:

$$x_{\text{norm}} = \frac{x - x_{\text{min}}}{x_{\text{max}} - x_{\text{min}}}$$

Dimensionality Reduction and Clustering

UMAP Projection

The UMAP projection in Figure 1A was computed using the umap-learn package (v0.5.3) [2]. UMAP (Uniform Manifold Approximation and Projection) is a dimensionality reduction technique that preserves both the local and global structure of high-dimensional data. Unlike linear methods like PCA, UMAP can capture complex nonlinear relationships, making it particularly suitable for visualising the intricate patterns in biological data. The algorithm works by first

constructing a high-dimensional graph representing the data's topological structure, then optimising a low-dimensional layout that preserves this structure as faithfully as possible. We applied UMAP to the integrated latent representations $Z \in \mathbb{R}^{n \times d}$ learned by CoMM-BIP to visualise how different effector conditions and genotypes organise in the reduced space.

Parameters used:

- `n_neighbours`: 15 (balances local vs. global structure)
- `min_dist`: 0.1 (controls cluster compactness)
- `metric`: cosine (suitable for high-dimensional biological data)
- `n_components`: 2 (for 2D visualisation)
- `random_state`: 42 (for reproducibility)

Functional Enrichment Analysis

Gene set enrichment was performed using GSEApy (v1.0.4) [3]. which implements the Gene Set Enrichment Analysis (GSEA) methodology. This approach determines whether members of a gene set tend to occur toward the top (or bottom) of a ranked gene list, where ranking is based on correlation with effector expression patterns. GSEA is particularly powerful because it detects subtle but coordinated changes in expression across entire pathways, rather than relying on arbitrary significance thresholds for individual genes [4]. For each effector-specific module, we tested enrichment against multiple databases to ensure comprehensive coverage of biological processes:

- Gene Ontology (GO) Biological Process terms
- KEGG pathways
- PlantCyc pathways (custom gene set)

Enrichment significance was calculated using the hypergeometric test with Benjamini-Hochberg FDR correction (threshold: FDR < 0.05). Values in Figure 1B-C represent $-\log_{10}(\text{FDR})$.

Hierarchical Clustering

Hierarchical clustering (Figure 1C, Figure 4C) was performed using SciPy (v1.9.0):

1. Distance matrix: Euclidean distance on $-\log_{10}(\text{FDR})$ values
2. Linkage method: Ward's method (`scipy.cluster.hierarchy.ward`)
3. Visualisation: Seaborn (v0.12.0) clustermap

Network Construction and Analysis

Cross-Modal Attention Networks (Figure 2F)

Networks were constructed from learned attention matrices A_{ij} following established practices for biological network analysis [5]. The attention mechanism in CoMM-BIP learns pairwise importance weights between features across different modalities, which naturally form a bipartite graph structure. By thresholding and symmetrising these weights, we obtain an undirected network where edge strengths represent the model's learned importance of cross-modal interactions. Network centrality measures (degree, betweenness) were calculated using standard graph algorithms to identify key integration hubs [6].

Networks were constructed from learned attention matrices A_{ij}:

1. Attention weights averaged across heads/layers using PyTorch (v1.13.0)
2. Matrix symmetrised: $\frac{A_{ij}+A_{ji}}{2}$
3. Edge threshold: $w > 0.8$ (retain top 20% of connections)
4. Node size: Degree centrality $= \sum_j w_{ij}$
5. Cross-Modality Relevance Score: $\text{Relevance}_i = \sum_{j \neq i} A_{ji}$

Network visualisation using NetworkX (v2.8.8) and Matplotlib (v3.5.3).

Effector-Target Interaction Networks (Figure 1E-F, 4F)

Molecular interaction networks integrate multiple evidence sources to provide a comprehensive view of effector targeting mechanisms. We combined computationally predicted interactions from STRING-db [6] with empirically learned attention weights from CoMM-BIP. This hybrid approach leverages both established biological knowledge and data-driven discoveries. STRING interactions include curated databases, experimental evidence, and computational predictions, providing a robust foundation that we enhance with model-specific insights from our multimodal analysis.

1. **Nodes:** Top 50 genes by attention weight from each effector module
2. **Edges:** STRING-db interactions (confidence > 0.7) + significant cross-modal correlations ($|r| > 0.6$, $p < 0.01$)
3. **Node size:** Absolute attention weight from effector
4. **Edge weight (Fig 4F):** $-\log_{10}(P_{\text{enrichment}})$

Statistical Analysis

Correlation Analysis

We employed both parametric (Pearson) and non-parametric (Spearman) correlation measures based on data distribution characteristics. Pearson correlation assumes linear relationships and normal distributions, making it suitable for normalised gene expression data [1]. Spearman's rank correlation is more robust to outliers and non-normal distributions, making it appropriate for phenotypic measurements that may not meet parametric assumptions [7]. Multiple testing correction using the Benjamini-Hochberg false discovery rate (FDR) procedure controls the expected proportion of false positives among significant results, which is crucial in high-dimensional biological data analysis.

- Pearson correlation: `scipy.stats.pearsonr`
- Spearman correlation: `scipy.stats.spearmanr` (for phenotypic data)
- Multiple testing correction: `statsmodels.stats.multitest.fdrcorrection` (v0.13.5)

Differential Analysis

For group comparisons, we selected statistical tests based on data characteristics and experimental design. ANOVA models are appropriate for normally distributed data with multiple groups, while Kruskal-Wallis tests serve as non-parametric alternatives when normality assumptions are violated. Post-hoc testing following significant omnibus tests identifies specific group differences while controlling for multiple comparisons. All statistical analyses were implemented using established scientific computing libraries to ensure computational accuracy and reproducibility.

- ANOVA: `scipy.stats.f_oneway` with `scikit-posthocs` (v0.7.0) for post-hoc tests
- Kruskal-Wallis: `scipy.stats.kruskal` for non-parametric tests

Model Implementation Details

Core Packages and Versions

- PyTorch: 1.13.0+cu116
- PyTorch Lightning: 1.8.0
- Scikit-learn: 1.1.0
- NumPy: 1.22.4
- Pandas: 1.5.3
- Matplotlib: 3.5.3
- Seaborn: 0.12.0
- SciPy: 1.9.0

Google Colab Environment

- Runtime: Python 3.9.16
- Hardware: Tesla T4 GPU (16 GB), 12.7 GB RAM
- Storage: Google Drive integration for data persistence

Reproducibility

- Random seeds fixed: 42 (Python, NumPy, PyTorch)
- Deterministic algorithms enabled where possible
- Complete notebook available: `https://github.com/Morillalab/CoMM-BIP`

References

[1] Ana Conesa, Pedro Madrigal, Sonia Tarazona, David Gomez-Cabrero, Alejandra Cervera, Andrew McPherson, Micha Wojciech Szczeniak, Daniel J Gaffney, Laura L Elo, Xuegong Zhang, et al. A survey of best practices for rna-seq data analysis. *Genome biology*, 17(1):1–19, 2016.

[2] Leland McInnes, John Healy, and James Melville. Umap: Uniform manifold approximation and projection for dimension reduction. *arXiv preprint arXiv:1802.03426*, 2018.

[3] Aravind Subramanian, Pablo Tamayo, Vamsi K Mootha, Sayan Mukherjee, Benjamin L Ebert, Michael A Gillette, Amanda Paulovich, Scott L Pomeroy, Todd R Golub, Eric S Lander, et al. Gene set enrichment analysis: a knowledge-based approach for interpreting genome-wide expression profiles. *Proceedings of the National Academy of Sciences*, 102(43):15545–15550, 2005.

[4] Guangchuang Yu, Li-Gen Wang, Yanyan Han, and Qing-Yu He. clusterprofiler: an r package for comparing biological themes among gene clusters. *Omics: a journal of integrative biology*, 16(5):284–287, 2012.

[5] Paul Shannon, Andrew Markiel, Owen Ozier, Nitin S Baliga, Jonathan T Wang, Daniel Ramage, Nada Amin, Benno Schwikowski, and Trey Ideker. Cytoscape: a software environment for integrated models of biomolecular interaction networks. *Genome research*, 13(11):2498–2504, 2003.

[6] Damian Szklarczyk, Annika L Gable, Katerina C Nastou, David Lyon, Rebecca Kirsch, Sampo Pyysalo, Nadezhda T Doncheva, Marc Legeay, Tao Fang, Peer Bork, et al. The string database in 2021: customizable protein–protein networks, and functional characterization of user-uploaded gene/measurement sets. *Nucleic acids research*, 49(D1):D605–D612, 2021.

[7] Trevor Hastie, Robert Tibshirani, Jerome H Friedman, and Jerome H Friedman. *The elements of statistical learning: data mining, inference, and prediction*, volume 2. Springer, 2009.
