# Supplementary material for: Multimodal learning reveals plants’ hidden sensory integration logic
Source: BMC Genomics. 2026 Feb 19;27:312. doi: 10.1186/s12864-026-12624-y (PMC13032346; doi:10.1186/s12864-026-12624-y)
Supplement: Supplementary file 2 — Supplementary Material 2: Table S1. Gene markers used for correlation analysis with UMAP axes. Figure S1. Functional annotation of effector-associated biological processes and protein domains. Enriched terms highlight iron/manganese ion homeostasis (e.g., transmembrane transport, vacuolar sequestration) mediated by VIT family transporters, alongside ATP-dependent RNA helicase activity (DEAD/DEAH box domains). Terms are clustered by functional similarity, reflecting coordinated roles in metal trafficking and RNA metabolism during effector activity. Figure S2. Unimodal data separability and model calibration analysis. (A, B, C) Calibration curve and confidence distribution demonstrate the model’s well-calibrated predictions, with 50% of cases falling in the high-confidence range ($0.75-0.92$) and no evidence of overconfidence. (D) Principal component analysis (PCA) of transcriptomic data shows clear separation of effector groups (GLOIN781 vs. GLOIN707) along PC1 (78.3% variance explained). (E, F) Phenomic and metabolomic profiles exhibit partial overlap between effectors (RiSP749, GLOIN781, OPF, GLOIN707), highlighting the need for multimodal integration. Figure S3. Extended analysis of phenotypic regression and embedding interpretability. (A-B) Trait-specific $R^2$(MSE) scores from phenotypic regression, highlighting stronger predictability for architectural traits. Corresponding mean squared errors reveal higher uncertainty in physiological traits such as anthocyanin accumulation. Perfor [file 12864_2026_12624_MOESM2_ESM.zip › TableS1.pdf]

Table 1: *
Supplementary Table S1. Gene markers used for correlation analysis with UMAP axes.

**Gene Symbol**	**Description**	**Correlation with** $UMAP_1$	**p-value**
JAZ1	Jasmonate ZIM-domain protein 1	-0.912	1.2×10^{-8}
AOC	Allene oxide cyclase	-0.885	3.4×10^{-7}
IAA9	Auxin/indole-3-acetic acid protein 9	-0.843	2.1×10^{-6}
MYC2	Transcription factor MYC2	-0.796	8.7×10^{-5}
OPR3	12-oxophytodienoate reductase 3	-0.782	1.4×10^{-4}
ARF7	Auxin response factor 7	-0.765	3.2×10^{-4}
SAUR50	SAUR family protein	-0.721	1.1×10^{-3}

**Gene Symbol**	**Description**	**Correlation with** $UMAP_2$	**p-value**
GLYI4	Lactoylglutathione lyase	-0.897	4.8×10^{-8}
GLYII2	Hydroxyacylglutathione hydrolase	-0.854	1.5×10^{-6}
VIT1	Vacuolar iron transporter 1	0.823	5.2×10^{-6}
FRO2	Ferric reduction oxidase 2	0.801	1.8×10^{-5}
NRAMP1	Nat. resist-assoc. macrophage prot 1	0.778	4.1×10^{-5}
IRT1	Iron-regulated transporter 1	0.745	2.3×10^{-4}

Note: Pearson correlation coefficients were calculated between normalised gene expression values ($\log_2(\text{TPM}+1)$) and UMAP coordinates. P-values were adjusted for multiple testing using the Benjamini-Hochberg (FDR) method. Genes are listed in order of decreasing absolute correlation strength for each UMAP axis.
