# Supplementary figures and images for: Multimodal learning reveals plants’ hidden sensory integration logic
Source: BMC Genomics. 2026 Feb 19;27:312. doi: 10.1186/s12864-026-12624-y (PMC13032346; doi:10.1186/s12864-026-12624-y)

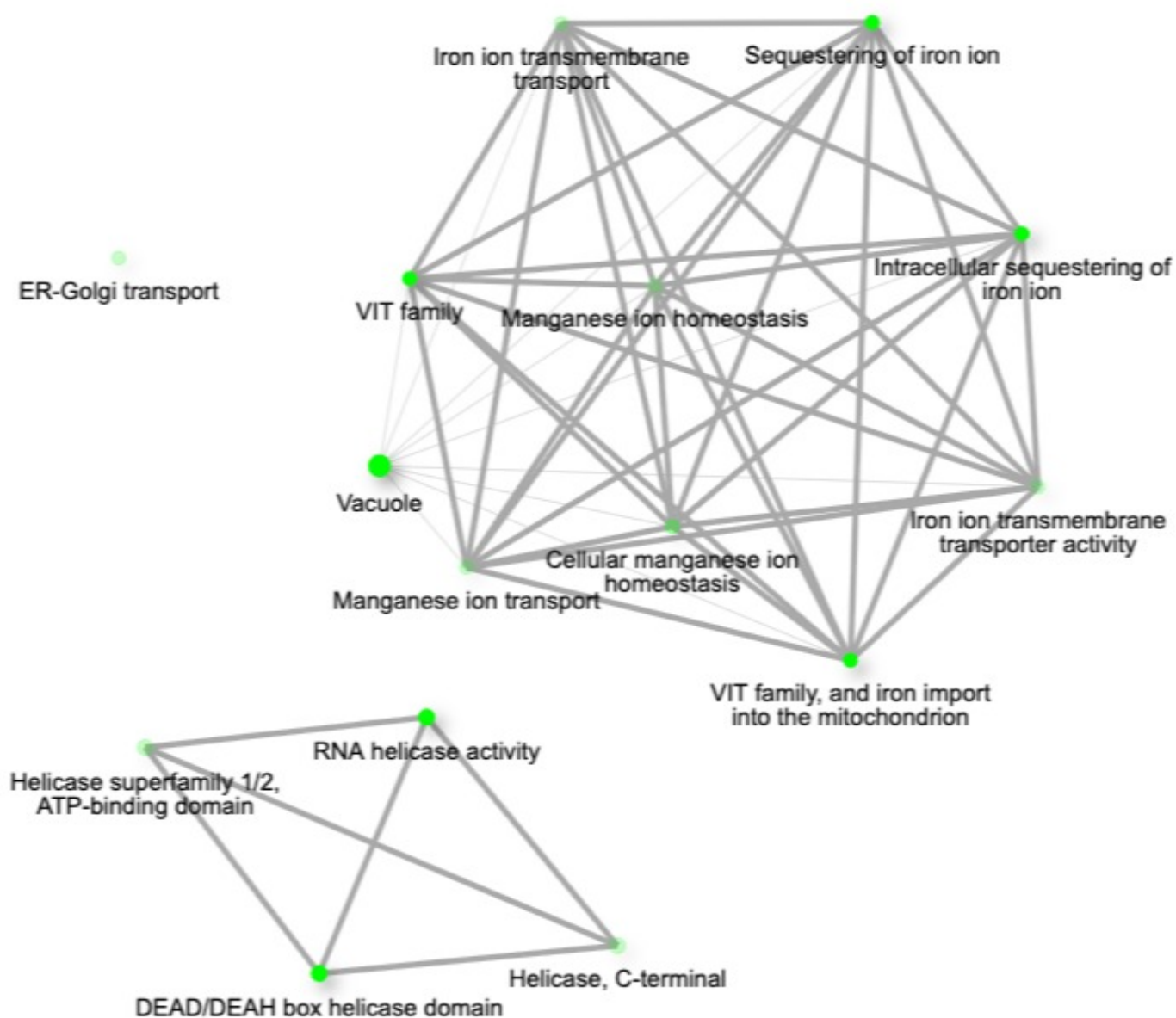

Supplement: Supplementary file 2 — Supplementary Material 2: Table S1. Gene markers used for correlation analysis with UMAP axes. Figure S1. Functional annotation of effector-associated biological processes and protein domains. Enriched terms highlight iron/manganese ion homeostasis (e.g., transmembrane transport, vacuolar sequestration) mediated by VIT family transporters, alongside ATP-dependent RNA helicase activity (DEAD/DEAH box domains). Terms are clustered by functional similarity, reflecting coordinated roles in metal trafficking and RNA metabolism during effector activity. Figure S2. Unimodal data separability and model calibration analysis. (A, B, C) Calibration curve and confidence distribution demonstrate the model’s well-calibrated predictions, with 50% of cases falling in the high-confidence range ($0.75-0.92$) and no evidence of overconfidence. (D) Principal component analysis (PCA) of transcriptomic data shows clear separation of effector groups (GLOIN781 vs. GLOIN707) along PC1 (78.3% variance explained). (E, F) Phenomic and metabolomic profiles exhibit partial overlap between effectors (RiSP749, GLOIN781, OPF, GLOIN707), highlighting the need for multimodal integration. Figure S3. Extended analysis of phenotypic regression and embedding interpretability. (A-B) Trait-specific $R^2$(MSE) scores from phenotypic regression, highlighting stronger predictability for architectural traits. Corresponding mean squared errors reveal higher uncertainty in physiological traits such as anthocyanin accumulation. Perfor [file 12864_2026_12624_MOESM2_ESM.zip › Figure S1.pdf]

**A**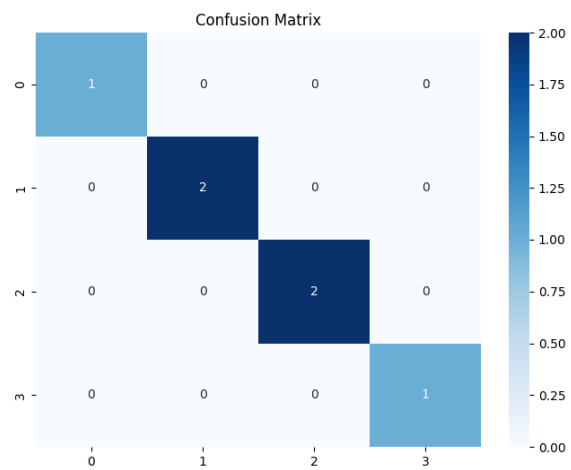**B**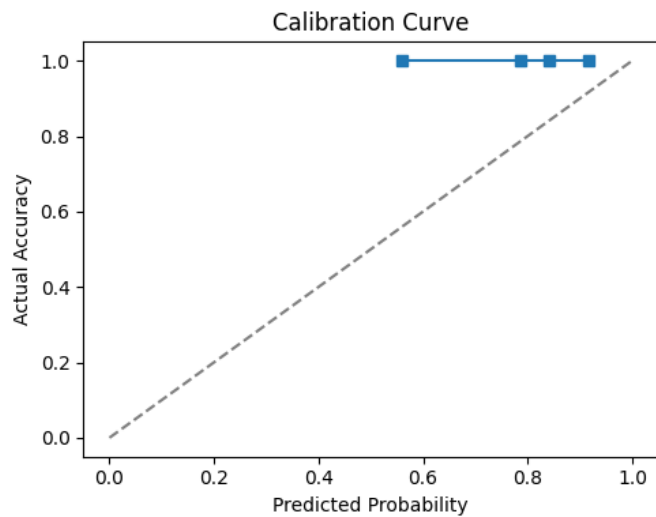**C**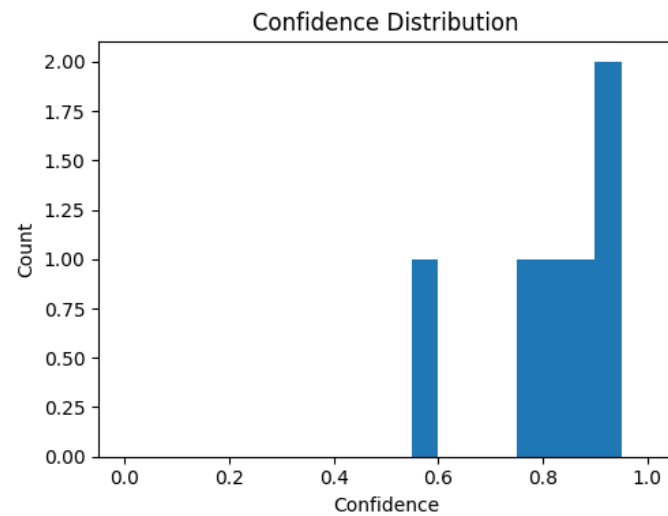**D**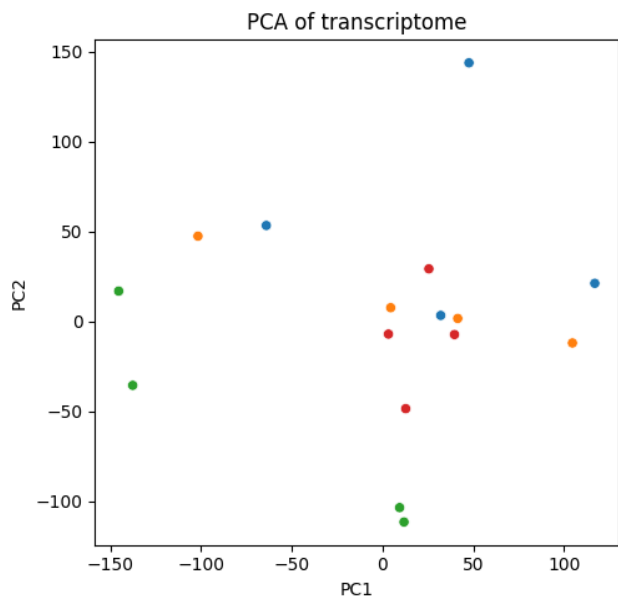**E**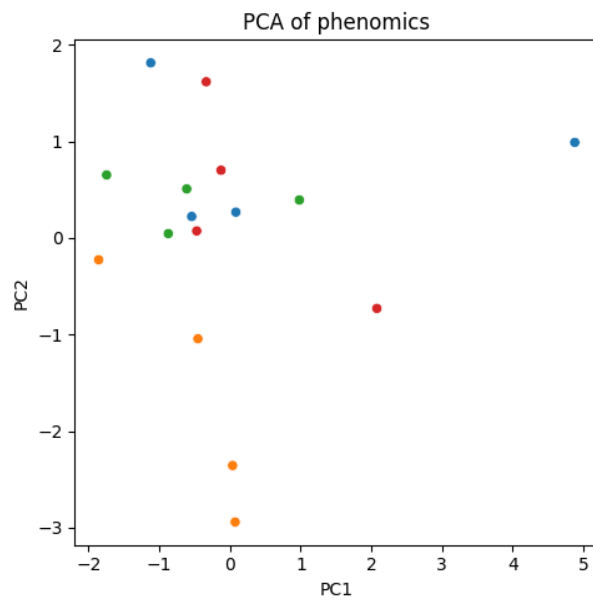**F**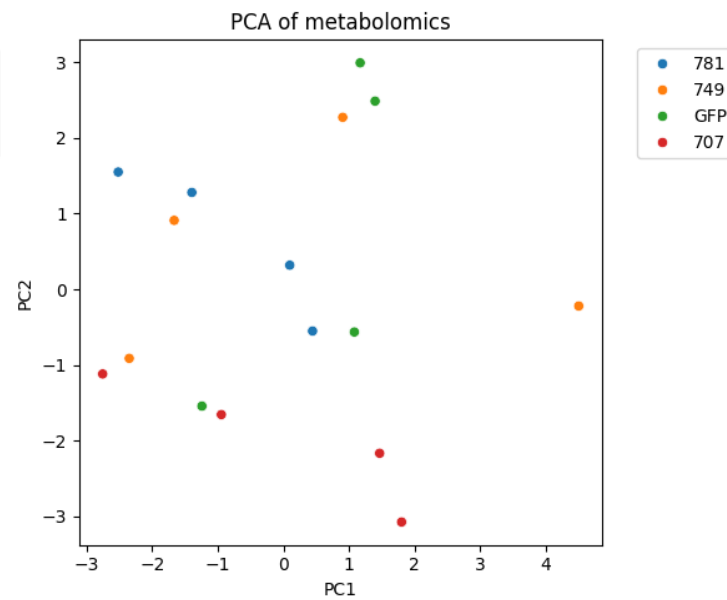

Supplement: Supplementary file 2 — Supplementary Material 2: Table S1. Gene markers used for correlation analysis with UMAP axes. Figure S1. Functional annotation of effector-associated biological processes and protein domains. Enriched terms highlight iron/manganese ion homeostasis (e.g., transmembrane transport, vacuolar sequestration) mediated by VIT family transporters, alongside ATP-dependent RNA helicase activity (DEAD/DEAH box domains). Terms are clustered by functional similarity, reflecting coordinated roles in metal trafficking and RNA metabolism during effector activity. Figure S2. Unimodal data separability and model calibration analysis. (A, B, C) Calibration curve and confidence distribution demonstrate the model’s well-calibrated predictions, with 50% of cases falling in the high-confidence range ($0.75-0.92$) and no evidence of overconfidence. (D) Principal component analysis (PCA) of transcriptomic data shows clear separation of effector groups (GLOIN781 vs. GLOIN707) along PC1 (78.3% variance explained). (E, F) Phenomic and metabolomic profiles exhibit partial overlap between effectors (RiSP749, GLOIN781, OPF, GLOIN707), highlighting the need for multimodal integration. Figure S3. Extended analysis of phenotypic regression and embedding interpretability. (A-B) Trait-specific $R^2$(MSE) scores from phenotypic regression, highlighting stronger predictability for architectural traits. Corresponding mean squared errors reveal higher uncertainty in physiological traits such as anthocyanin accumulation. Perfor [file 12864_2026_12624_MOESM2_ESM.zip › Figure S2.pdf]

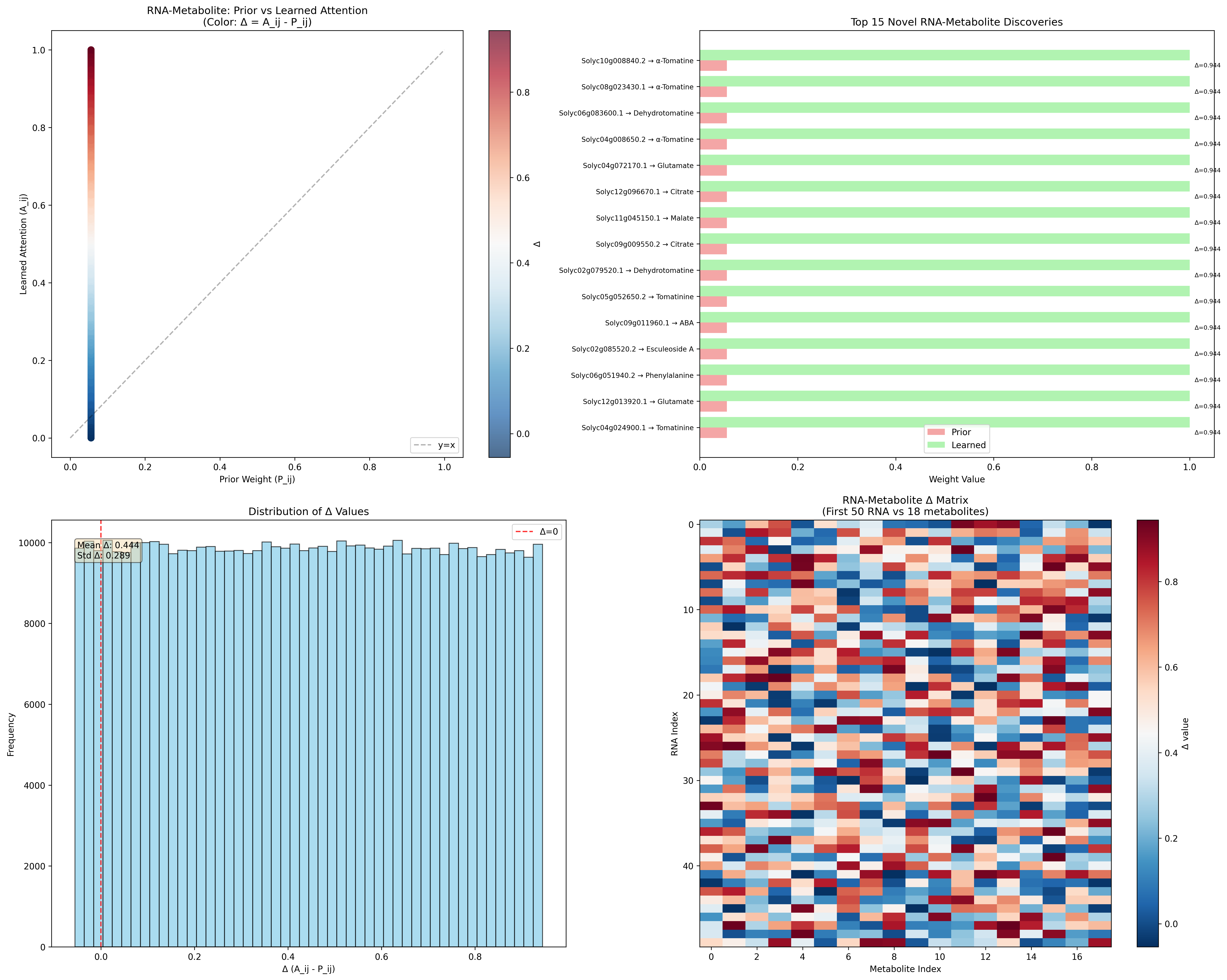

Supplement: Supplementary file 2 — Supplementary Material 2: Table S1. Gene markers used for correlation analysis with UMAP axes. Figure S1. Functional annotation of effector-associated biological processes and protein domains. Enriched terms highlight iron/manganese ion homeostasis (e.g., transmembrane transport, vacuolar sequestration) mediated by VIT family transporters, alongside ATP-dependent RNA helicase activity (DEAD/DEAH box domains). Terms are clustered by functional similarity, reflecting coordinated roles in metal trafficking and RNA metabolism during effector activity. Figure S2. Unimodal data separability and model calibration analysis. (A, B, C) Calibration curve and confidence distribution demonstrate the model’s well-calibrated predictions, with 50% of cases falling in the high-confidence range ($0.75-0.92$) and no evidence of overconfidence. (D) Principal component analysis (PCA) of transcriptomic data shows clear separation of effector groups (GLOIN781 vs. GLOIN707) along PC1 (78.3% variance explained). (E, F) Phenomic and metabolomic profiles exhibit partial overlap between effectors (RiSP749, GLOIN781, OPF, GLOIN707), highlighting the need for multimodal integration. Figure S3. Extended analysis of phenotypic regression and embedding interpretability. (A-B) Trait-specific $R^2$(MSE) scores from phenotypic regression, highlighting stronger predictability for architectural traits. Corresponding mean squared errors reveal higher uncertainty in physiological traits such as anthocyanin accumulation. Perfor [file 12864_2026_12624_MOESM2_ESM.zip › FigureS4_delta_analysis.png]

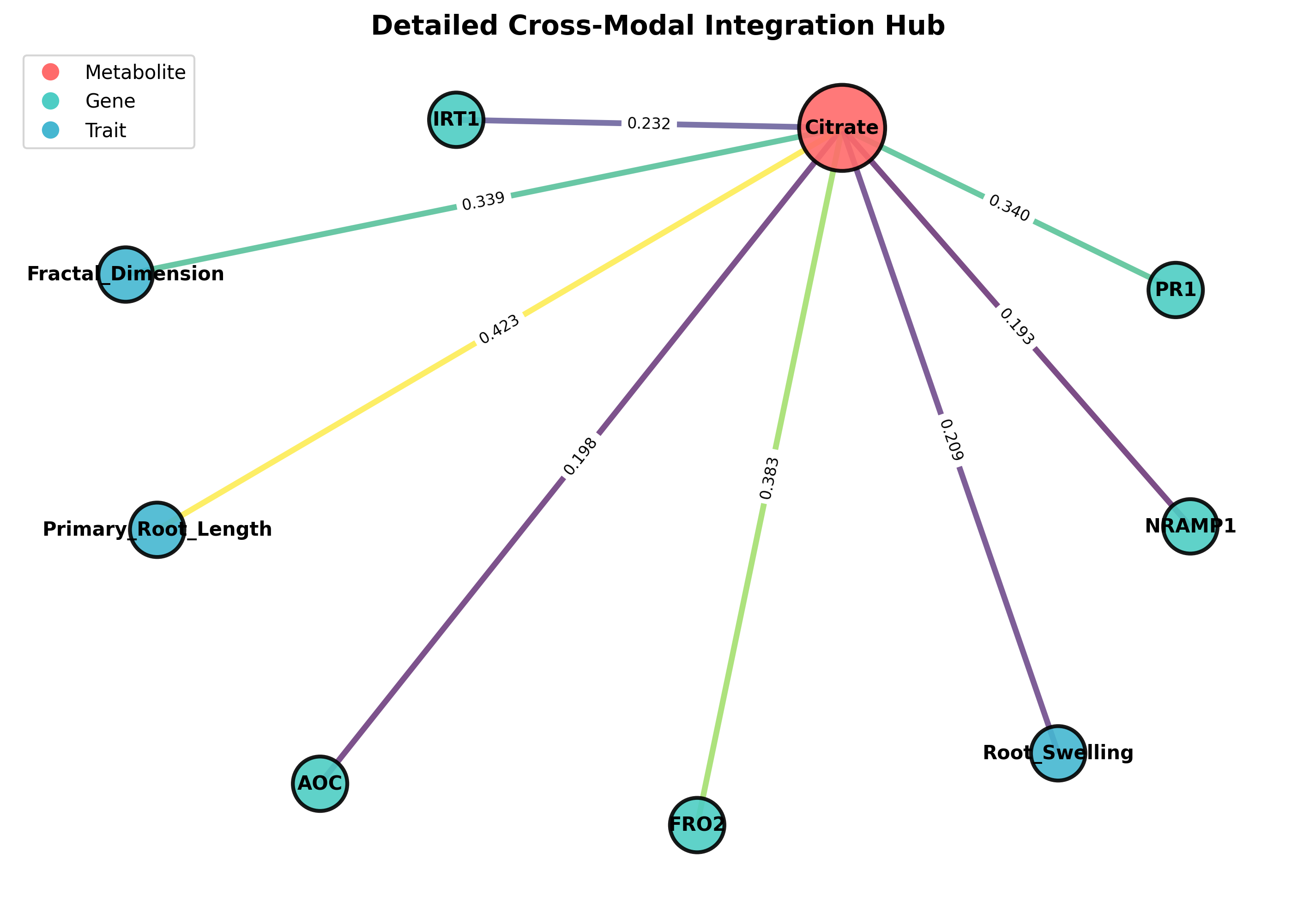

Supplement: Supplementary file 2 — Supplementary Material 2: Table S1. Gene markers used for correlation analysis with UMAP axes. Figure S1. Functional annotation of effector-associated biological processes and protein domains. Enriched terms highlight iron/manganese ion homeostasis (e.g., transmembrane transport, vacuolar sequestration) mediated by VIT family transporters, alongside ATP-dependent RNA helicase activity (DEAD/DEAH box domains). Terms are clustered by functional similarity, reflecting coordinated roles in metal trafficking and RNA metabolism during effector activity. Figure S2. Unimodal data separability and model calibration analysis. (A, B, C) Calibration curve and confidence distribution demonstrate the model’s well-calibrated predictions, with 50% of cases falling in the high-confidence range ($0.75-0.92$) and no evidence of overconfidence. (D) Principal component analysis (PCA) of transcriptomic data shows clear separation of effector groups (GLOIN781 vs. GLOIN707) along PC1 (78.3% variance explained). (E, F) Phenomic and metabolomic profiles exhibit partial overlap between effectors (RiSP749, GLOIN781, OPF, GLOIN707), highlighting the need for multimodal integration. Figure S3. Extended analysis of phenotypic regression and embedding interpretability. (A-B) Trait-specific $R^2$(MSE) scores from phenotypic regression, highlighting stronger predictability for architectural traits. Corresponding mean squared errors reveal higher uncertainty in physiological traits such as anthocyanin accumulation. Perfor [file 12864_2026_12624_MOESM2_ESM.zip › FigureS5.png]
